# circ_0004140 promotes LUAD tumor progression and immune resistance through circ_0004140/miR-1184/CCL22 axis

**DOI:** 10.1038/s41420-022-00983-w

**Published:** 2022-04-08

**Authors:** Yanyan Liu, Haodong Zhang, Wangli Zhang, Lanxin Xiang, Zhucheng Yin, Hongli Xu, Ping Lu, Yifei Ma, Lingyi Xiong, Xiangchen Zhang, Xin Liang, Jing Luo, Xinjun Liang

**Affiliations:** 1 Division of Nephrology, Tongji Hospital, Tongji Medical College, Huazhong University of Science and Technology, 1095 Jiefang Ave, 430030 Wuhan, Hubei P. R. China; 2 School of life science and technology, Huazhong Agricultural University, 430070 Wuhan, Hubei P. R. China; 3 Department of Medical Oncology, Hubei Cancer Hospital, Tongji Medical College, Huazhong University of Science and Technology, No 116 Zhuodaoquan South Load, Hongshan District, 430079 Wuhan, Hubei P. R. China; 4 Institute of Reproductive Health, Center for Reproductive Medicine, Tongji Medical College, Huazhong University of Science and Technology, Wuhan, China

**Keywords:** Non-small-cell lung cancer, Tumour biomarkers

## Abstract

Lung adenocarcinoma (LUAD) is a highly prevalent cancer with high mortality. Immune resistance and tumor metastasis are the pivotal factors for the promotion of LUAD. CircRNAs have been revealed a crucial pre-clinical diagnostic and therapeutic potentials in LUAD. Herein, we identify a novel circRNA (circ_0004140), derived from the oncogene YAP1, which is up-regulated in LUAD. The high expression of circ_0004140 is correlated with poor prognosis and CTL cells dysfunction in LUAD patients. Knockdown of circ_0004140 regulated LUAD cells proliferation, migration, and apoptosis. Mechanistically, circ_0004140 served as a sponge of miR-1184 targeting C-C motif chemokine ligand 22(CCL22). Overexpression of CCL22 reversed the inhibitory effect induced by si-circ_0004140 on cells proliferation and migration. Moreover, we also revealed that elevated circ_ooo4140 was related to cytotoxic lymphocyte exhaustion, and a combination therapy of C-021 (CCL22/CCR4 axis inhibitor) and anti-PD-1 attenuated LUAD promotion and immune resistance. In conclusion, circ_0004140 may drive resistance to anti-PD-1 immunotherapy, providing a novel potential therapeutic target for LUAD treatment.

## Introduction

Lung cancer is the world’s leading cause of cancer-related mortality and approximately 40% of diagnosed cases are lung adenocarcinomas (LUAD) [1]. Immune checkpoints blockers (ICBs) including programmed cell death protein 1(PD-1) and cytotoxic T-lymphocyte-associated protein 4 (CTLA-4) have achieved clinical improvements on LUAD treatment [2, 3]. However, plenty of LUAD patients acquired immune resistance after several cycles of immunotherapy and the 5-year overall survival rate of LUAD patients is still unsatisfactory [4, 5]. The underlying molecular mechanisms of LUAD tumorigenesis, metastasis, and resistance to immunotherapy still remain unclear. Hence, a further investigation of the biological process and molecular mechanisms of LUAD promotion are urgently needed.

Circular RNAs (circRNAs) are a novel type of the noncoding RNAs characterized by covalent-closed loop structures that lack of 5′ to 3′ polyadenylated tails, which are formed via backsplicing [6]. The expression of circRNAs is evolutionarily conserved and sometimes much higher than their host genes [7]. Accumulating evidence has revealed that circRNAs play important roles in both physiological and pathological processes such as tumorigenesis, distant metastasis, and immune escape in various cancers [8, 9]. CircRNAs can function as a molecule sponge for targeting miRNAs, thus promoting the expression of downstream targeting mRNAs [10]. Circ_ENO1 was significantly up-regulated in LUAD tissue and cells. And circ_ENO1 increased ENO1 expression by sponging miR-22-3p, resulting in enhanced glycolysis and EMT process [11]. In addition, circular RNA cMras was reported that inhibiting tumorigenesis and metastasis by attenuating miR-567 expression and further promoting PTPRG expression [12]. Although the molecular mechanisms of dysregulated circRNAs in LUAD have been extensively investigated, the function and underlying mechanism of circRNAs in immune resistance in LUAD are still not fully understood.

C-C motif chemokine ligand 22 (CCL22) is a secreted protein that exerts chemotactic activity for monocytes, dendritic cells, and natural killer cells [13]. The cognate receptor of CCL22 is CCR4, a transmembrane protein expressed predominantly and constitutively by regulatory T cells [14]. Previous research indicated that CCL22 is abundantly expressed that induces tumorigenesis and tumor progression in different types of cancers such as cervical cancer [15], breast cancer [16], and ovarian carcinoma [17]. A recent study demonstrated that the expression of CCL22 in dendritic cells (DC) induced the interaction and contact with Treg cells via the CCR4 receptor [18]. Vaccination of CCL22-deficient mice resulted in excessive T cell immune responses, prolonged survival, and enhanced susceptibility to inflammatory reaction [19].

In this study, we identified a novel circRNA (circ_0004140) derived from oncogene YAP1, which was remarkably up-regulated in LUAD tissues and cell lines. And the dysregulated circ_0004140 was correlated with poor prognosis and CTL cell dysfunction in LUAD patients. Mechanistically, elevated circ_0004140 promoted CCL22 expression to contribute LUAD progression and induce immune resistance via sponging miR-1184. Our findings suggested that circ_0004140 might act as a novel promising therapeutic target for LUAD treatment.

## Results

### Circ_0004140 is relatively up-regulated in LUAD

**Fig. 1:**
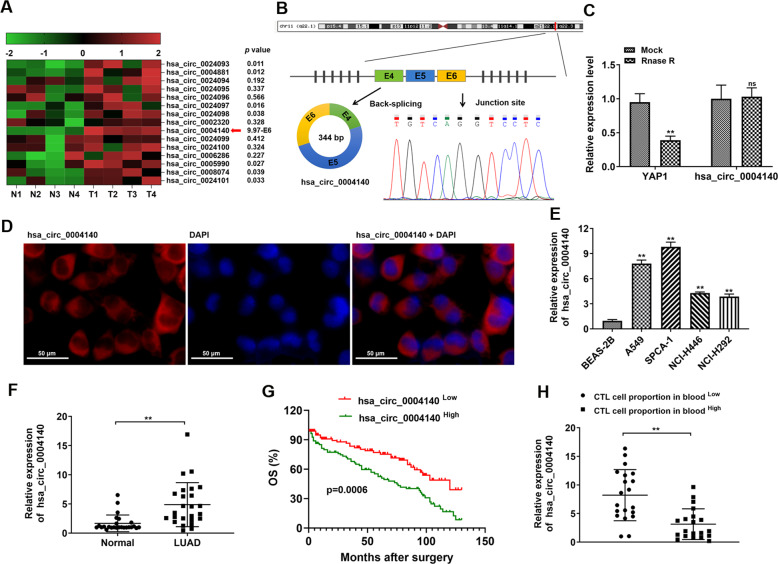
circ_0004140 is up-regulated in LUAD. **A** Heat map representing expression of 15 circRNAs derived from YAP1 in 4 pairs of LUAD tissues and adjacent normal tissues detected by RT-PCR analysis. Circ_0004140 was the most significantly up-regulated in LUAD samples. **B** Schematic illustration indicated circ_0004140 was generated from exons 4–6 of YAP1 gene. Sanger sequencing revealed the junction site of circ_0004140. **C** Resistance of circ_0004140 to RNase R was verified through qRT-PCR after RNase R digestion (*n* = 3). **D** The cellular distribution of circ_0004140 was analyzed by fluorescence in situ hybridization (FISH) (*n* = 3). Red indicates circ_0004140. Nuclei were stained with DAPI. Scale bar=50μm. **E**, **F** circ_0004140 was up-regulated in LUAD cell lines (A549, SPCA-1, NCI-H446, and NCI-H292) (E) compared with BEAB-2B cells (*n* = 3) and in LUAD clinical samples (F) (*n* = 25). **G** Kaplan–Meier analysis of overall survival in LUAD patients with high circ_0004140 expression (*n* = 80) and low circ_0004140 expression (*n* = 80). **H** 40 LUAD patients were divided into high and low groups based on CTL cell proportion in blood. The expression of circ_0004140 was determined in each group through RT-PCR analysis (n-20). *p < 0.05, **p < 0.01, ns non-significant. All the data are representative of at least three independent experiments and presented as the means ± SD.

Previous studies have revealed that YAP1 is up-regulated in different types of cancers, which is corrected with tumorigenesis and tumor progression [20, 21]. Therefore, we detected 15 circRNAs derived from YAP1 gene in circBase database from 4 paired LUAD clinical samples. The results showed that the expression of circ_0004140 was the most significantly increased in LUAD samples compared with matched normal samples (Fig. 1A). By examining the UCSC database, we examined that the 344-bp circ_0004140 was generated by circularization of exon 4, exon 5 and exon 6 of YAP1 gene with the position of chr11:102056748-102080295 and the sequence of splice junction was identified through Sanger sequencing (Fig. 1B). Besides, the RT-PCR results determined that RNase R degraded the linear transcript of YAP1, while the circular transcript of circ_0004140 was resistant to digestion with RNase R, implying that circ_0004140 was circular (Fig. 1C). Besides, we found that circ_0004140 expression was stable with actinomycin D treatment at different times (Fig. S1). RNA FISH analysis revealed that circ_0004140 was mainly located in the cytoplasm (Fig. 1D). Moreover, RT-PCR analysis showed that circ_0004140 was significantly up-regulated in LUAD cell lines (A549, SPCA-1, NCI-H446, and NCI-H292) (Fig. 1E) and in 25 LUAD clinical samples compared with adjacent normal tissues (Fig. 1F). A549 and SPCA-1 cell lines were selected for further experiments. Next, Kaplan–Meier analysis showed that LUAD patients with high circ_0004140 expression (*n* = 80) had shorter overall survival than cases with low circ_0004140 expression (*n* = 80) (Fig. 1G). In addition, we explored the relationship between clinicopathological characteristics and circ_0004140 expression of 40 LUAD patients (Table 1). The results revealed that LUAD patients with high circ_0004140 expression have lower CTL cell proportions in blood (Fig. 1H). Taken together, circ_0004140 might be a critical promoter of LUAD.

**Table 1:** Clinical characteristics of LUAD patients according to CTL cell proportion.

Feature	CTL cell proportion Low	High	*p* value
Age			
<50	29	31	0.722
≥50	11	9	
Gender			
Male	25	22	0.616
Female	15	18	
Tumor size (cm)			
<5	11	27	0.001^**^
≥5	29	13	
Tumor differentiation			
I/II	13	24	0.016^*^
III/IV	27	16	
Lymphatic metastasis			
Yes	28	15	0.007^**^
No	12	25	
Distant metastasis			
Yes	24	13	0.002^**^
No	16	27	

*p < 0.05, **p < 0.01 in statistics.

### Circ_0004140 promotes the proliferation, migration, and apoptosis in LUAD cells

**Fig. 2:**
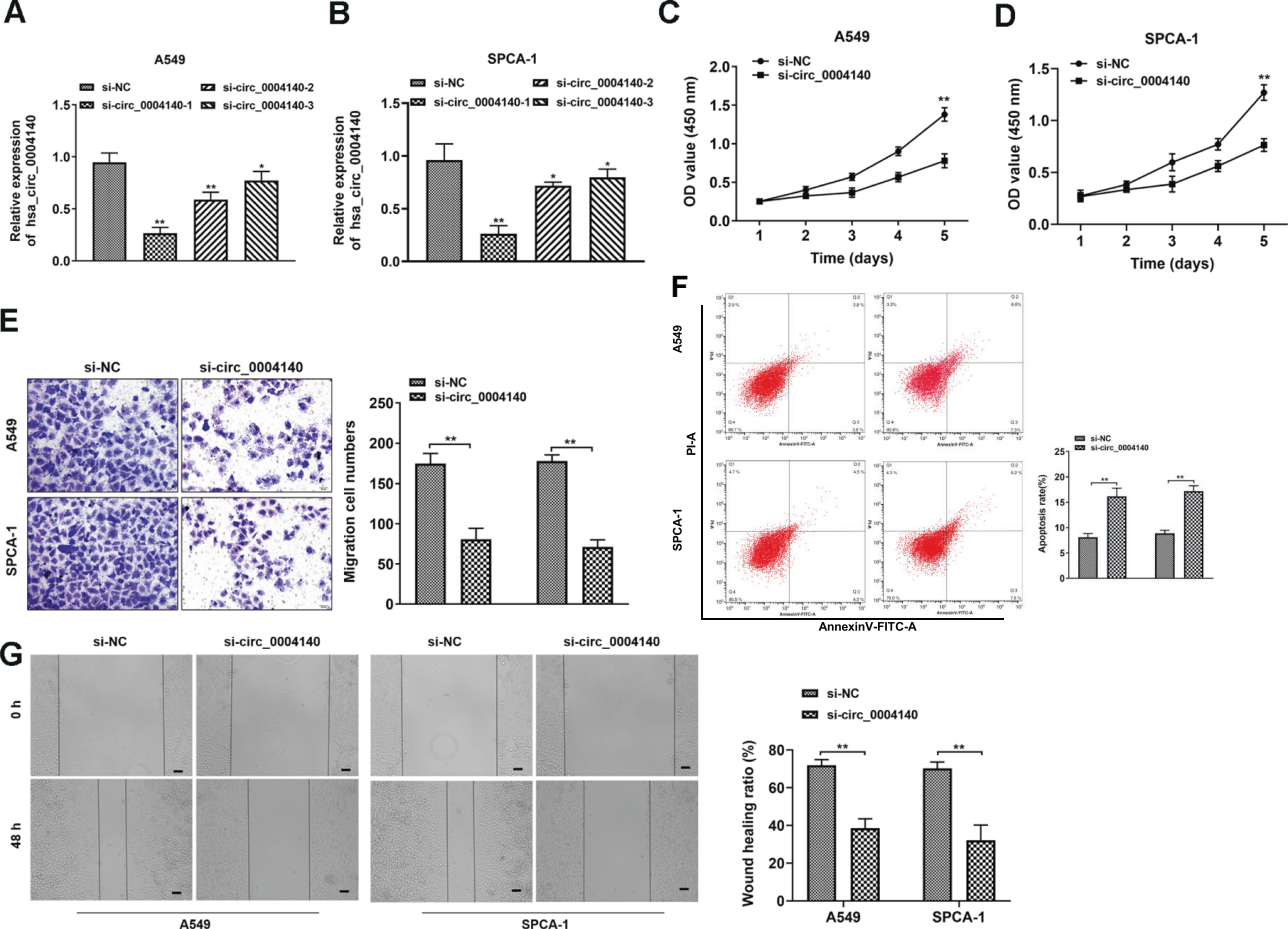
circ_0004140 promotes the proliferation, migration and apoptosis in LUAD cells. **A**, **B** Three different siRNAs were used to determine the most knockdown efficiency in A549 cells (**A**) and SPCA-1 cells (**B**) measured by RT-PCR analysis (*n* = 3). **C**, **D** Effect of circ_0004140 knockdown on cell viability in A549 cells (**C**) and SPCA-1 cells (**D**) determined by CCK-8 assay (*n* = 3). **E** The effect of circ_0004140 on cell invasion was evaluated by transwell assay(*n* = 3). Scale bar = 20 μm. **F** The apoptosis of LUAD cells was measured by staining with Annexin V/PI, followed by FACS analysis (*n* = 3). The abscissa indicated the number of cells positive for Annexin V, and the ordinate indicated the number of cells positive for PI. **G** The migrated ability of A549 and SPCA-1 cells was assessed through wound healing assay (*n* = 3). Scale bar = 20 μm.*p < 0.05, **p < 0.01, ns non-significant. All the data are representative of at least three independent experiments and presented as the means ± SD.

To further investigate the functional effects of circ_0004140 in vitro, we designed and synthesized three siRNAs targeting the circ_0004140 junction site and transfected the siRNA or negative control into A549 and SPCA-1 cells. The transfection efficiency was validated via RT-PCR analysis (Fig. 2A, B). CCK-8 assay was performed to explore the proliferation of A549 and SPCA-1 cells. A notable inhibition of cell proliferation after circ_0004140 siRNA transfection was observed (Fig. 2C, D). Moreover, cell invasion ability was examined through transwell assay. As shown in Fig. 2E, knockdown of circ_0004140 effectively suppressed the invasion ability of LUAD cells. Besides, flow cytometry analysis revealed that circ_0004140 inhibition remarkably promoted cell apoptosis (Fig. 2F). Furthermore, wound healing assay was applied to confirm that the cell migration was inhibited after circ_0004140 siRNA transfection compared to si-NC group (Fig. 2G).

### Circ_0004140 functions as an efficient miR-1184 sponge in LUAD cells

**Fig. 3:**
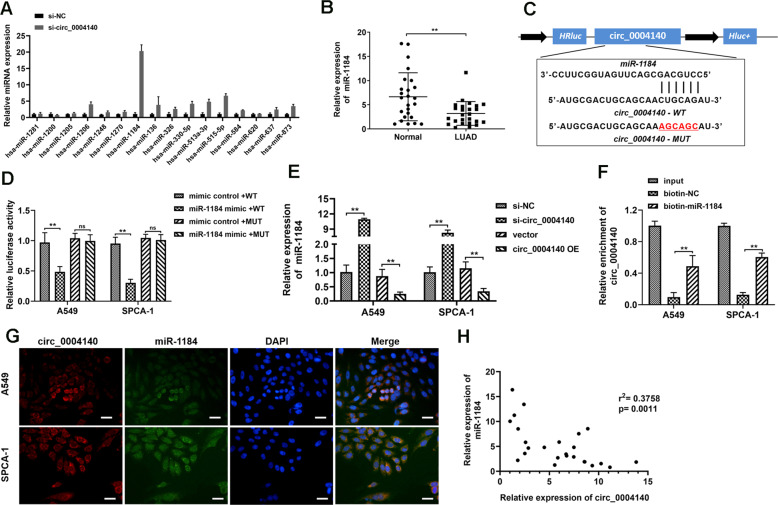
circ_0004140 functions as an efficient miR-1184 sponge in LUAD cells. **A** The expression of 16 potential targeting miRNAs of circ_0004140 was detected by RT-PCR analysis in A549 cells after circ_0004140 siRNA or NC transfection (*n* = 3). **B** RT-PCR analysis of miR-1184 expression in LUAD tissue samples (*n* = 25). **C** The putative binding site between circ_0004140 and miR-1184 was predicted through Starbase V3.0. **D** Luciferase reporter assay was applied to examine the direct interaction between circ_0004140 and miR-1184 (*n* = 3). **E** The expression of miR-1184 was measured by RT-PCR analysis in A549 and SPCA-1 cells after transfection with circ_0004140 overexpression plasmids (circ_0004140 OE), si-circ_0004140 or the control (*n* = 3). **F** RNA-pulldown assay was carried out in A549 and SPCA-1 cells to confirm the interaction between circ_0004140 and miR-1184 (*n* = 3). **G** RNA fluorescence in situ hybridization (RNA-FISH) determined the subcellular localization of circ_0004140 and miR-1184 in A549 and SPCA-1 cells (*n* = 3). Scale bar=50μm. **H** The Pearson correlation analysis was applied to reveal the negative correlation between circ_0004140 and miR-1184 in 25 paired LUAD tissues. *p < 0.05, **p < 0.01, ns non-significant. All the data are representative of at least three independent experiments and presented as the means ± SD.

Studies have determined that circRNAs sponge miRNAs to participate in the regulation of cell functions [22]. Therefore, bioinformatics analysis (circular RNA interactome) was screened to predict the potential targeting miRNAs of circ_0004140 and 16 miRNAs were predicted. Then, RT-PCR assay was performed to determine the expression of the series of miRNAs after circ_0004140 siRNA transfection. It was found that miR-1184 showed highest alteration (Fig. 3A). The RT-PCR analysis results determined that miR-1184 was remarkably down-regulated in clinical LUAD tissues compared to the adjacent normal tissues (Fig. 3B). Bioinformatic analysis showed the miR-1184 binding site with circ_0004140 (Fig. 3C). WT and MUT circ_0004140 were constructed for the Luciferase reporter assay. And the findings indicated that miR-1184 mimics only effectively inhibited the luciferase activity of the circ_0004140-WT compared with the other groups. (Fig. 3D). Besides, we constructed circ_0004140 overexpression plasmids and the overexpression efficiency were shown in Fig. S2. Circ_0004140 overexpression plasmids and siRNA were transfected into A549 and SPCA-1 cells, respectively. RT-PCR results showed that up-regulation of circ_0004140 obviously inhibited the expression of miR-1184 while down-regulation of circ_0004140 promoted miR-1184 expression (Fig. 3E). To further confirm the interaction of circ_0004140 and miR-1184, RNA pulldown was carried out in A549 and SPCA-1 cells. The results revealed that biotin-labeled miR-1184 enriched circ_0004140, thus indicating the binding interaction of circ_0004140 and miR-1184 (Fig. 3F). In addition, the FISH assay determined that circ-0004140 was co-localized in cytoplasm with miR-1184 in A549 and SCPCA-1 cells (Fig. 3G). Moreover, the Pearson analysis showed the negative correlation between circ_0004140 and miR-1184 (Fig. 3H).

### MiR-1184 directly targets CCL22 in LUAD cells

**Fig. 4:**
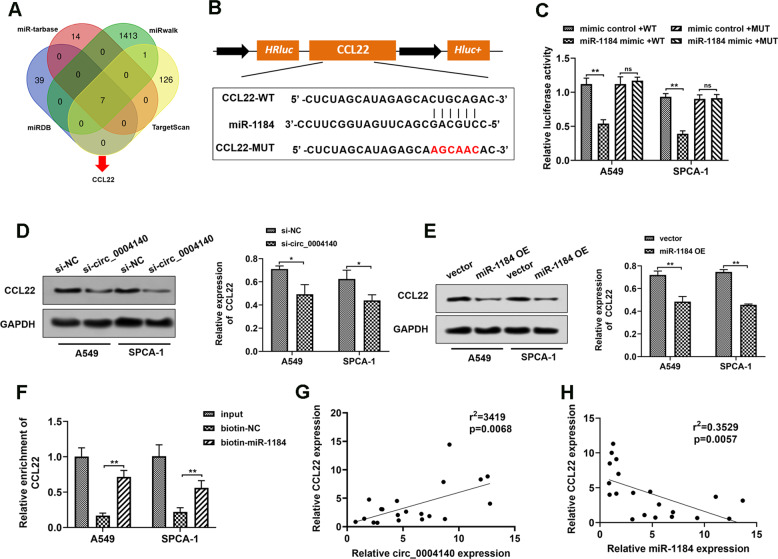
miR-1184 directly targets CCL22 in LUAD cells. **A** Bioinformatic analysis of the potential target genes of miR-1184 through miR-tarbase, miRwalk, miRDB and TargetScan. **B** Schematic diagram illustrated the potential binding sites between miR-1184 and CCL22. **C** Luciferase reporter assay unveiled the direct interaction within miR-1184 mimics and CCL22 mRNA wild-type (WT) or mutant (MUT) plasmids (*n* = 3). **D** Western Blot assay was carried out to investigate the CCL22 expression in A549 and SPCA-1 cells after transfection with si-circ_0004140 or NC (*n* = 3). **E** Western Blot assay was applied to examine the CCL22 expression in A549 and SPCA-1 cells after transfection with miR-1184 overexpression plasmids or blank vector (*n* = 3). **F** RNA pulldown analysis indicated the interaction between miR-1184 and CCL22 (*n* = 3). **G** A positive correlation between circ_0004140 and CCL22 mRNA expression was observed in 20 LUAD clinical samples. **H** A negative correlation between miR-1184 and CCL22 mRNA expression was confirmed in 20 LUAD samples. *p < 0.05, **p < 0.01, ns non-significant. All the data are representative of at least three independent experiments and presented as the means ± SD.

To elucidate the precise mechanism underlying the biological role induced by circ_0004140, we predicted the downstream target genes of miR-1184 from miRDB, miR-tarbase, miRwalk and TargetScan database (Fig. 4A). Based on the bioinformatic analysis, CCL22 was found as the putative target gene. Furthermore, to identify the interaction between miR-1184 and CCL22, we cloned the WT and MUT CCL22-3′UTR mRNA and performed luciferase reporter assay (Fig. 4B). The results revealed that miR-1184 mimics only markedly inhibited the luciferase intensity of co-transfection with WT CCL22-3′UTR compared with other groups (Fig. 4C). Besides, Western blot assay was carried out and the results showed that knockdown of circ_0004140 or overexpression of miR-1184 inhibited the expression of CCL22 (Fig. 4D, E). RNA pulldown assay was applied to further explore the interaction between miR-1184 and CCL22. As indicated by the findings, biotin-miR-1184 highly enriched CCL22 in LUAD cells. (Fig. 4F). Additionally, Pearson analysis revealed the significant negative correlation between miR-1184 and CCL22, and a positive correlation between circ_0004140 and CCL22 (Fig. 4G, H).

### Elevated CCL22 reverses circ_0004140 knockdown-induced biological functions

**Fig. 5:**
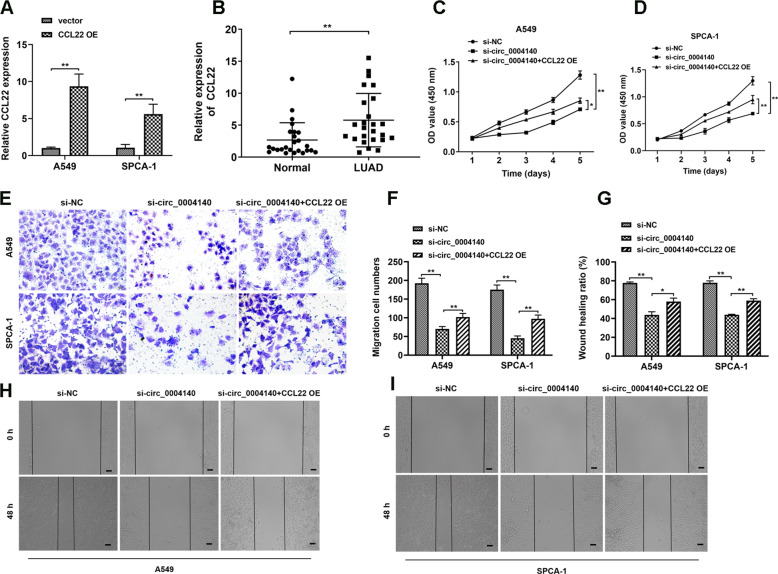
Elevated CCL22 reverses circ_0004140 knockdown-induced biological functions. **A** The overexpression efficiency was detected via RT-PCR analysis in A549 and SPCA-1 cells after transfection with CCL22 overexpression plasmid or blank vector (*n* = 3). **B** RT-PCR analysis was used to examine the CCL22 expression in 25 paired LUAD clinical samples. **C**, **D** The cell viability of A549 (**C**) and SPCA-1 (**D**) cells was determined by CCK-8 assay after co-transfection with si-circ_0004140 and CCL22 overexpression plasmids (*n* = 3). **E**, **F** Rescue transwell experiments were performed to validated cell invasion ability in co-transfection with si-circ_0004140 and CCL22 overexpression plasmids (*n* = 3). Scale bar=20 μm. **G–I)** The wound healing assay was carried out to assess the migration ability of A549 and SPCA-1 cells under different treatment (*n* = 3). Scale bar=20μm. *p < 0.05, **p < 0.01, ns non-significant. All the data are representative of at least three independent experiments and presented as the means ± SD.

To further examine the functions of CCL22 in LUAD, we constructed CCL22 overexpression plasmids and the overexpression efficiency was verified in A549 and SPCA-1 cells (Fig. 5A). Through RT-PCR analysis, we found that CCL22 expression was obviously up-regulated in LUAD clinical samples compared with paired normal tissues (Fig. 5B). CCK-8, transwell and wound healing assay were applied to explore the functional role of CCL22. The findings revealed that CCL22 overexpression remarkably reversed the cell proliferation, invasion, and migration ability that induced by circ_0004140 inhibition (Fig. 5C–I). These results indicated that up-regulation of CCL22 reversed the circ_0004140-induced function and promoted malignant phenotypes in LUAD.

### Circ_0004140 is related to cytotoxic T lymphocyte exhaustion and immunotherapy effect in vivo

**Fig. 6:**
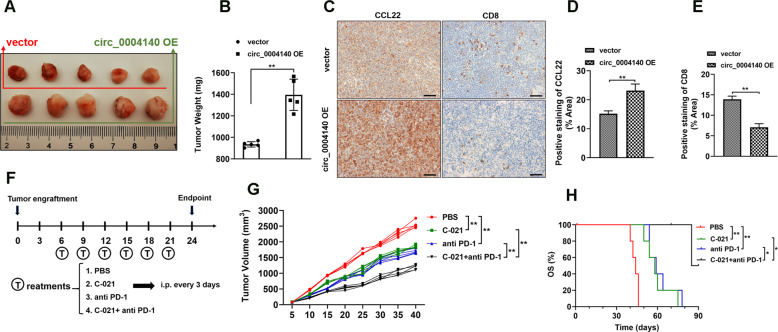
circ_0004140 is related to cytotoxic T lymphocyte exhaustion and immunotherapy effect in vivo. The morphology of the tumor xenografts (**A**) and the weight (**B**) of xenograft tumors formed by subcutaneous injection of LUAD cells stably transfected with circ_0004140 overexpression lentiviruses or control vector (*n* = 5). **C**–**E** IHC staining was utilized to detect the CCL22 expression (**C**, **D**) and CTL numbers (**C**, **E**) in xenograft tumor tissues (*n* = 3). Scale bar=100μm. **F** Schematic illustration of xenograft tumor treatment. Tumor-bearing mice were treated on indicated days with C-021 (CCL22 inhibitor), or anti-PD-1, or combination administration (*n* = 5). **G** Tumor growth was monitored through measuring tumor volume (*n* = 5). **H** Kaplan–Meier survival curve of C-021 or anti-PD1 or combination-treated xenograft mice (*n* = 5). *p < 0.05, **p < 0.01, ns non-significant. All the data are representative of at least three independent experiments and presented as the means ± SD.

CCL22 recruits Treg cells in the tumor microenvironment (TME) and treg cells could competitively bind with cytotoxic lymphocyte (CTL), thereby inhibiting the proliferation of CTL and contributing to immune suppression [23, 24]. To evaluate the influence of circ_0004140 on the proliferation of CTL, we established a xenograft model by injecting A549 cells transfected with circ_0004140 overexpression lentiviruses to the mice. The findings indicated that the circ_0004410 up-regulation notably promoted the tumor growth compared to the control group (Fig. 6A, B). Moreover, we found that CCL22 expression of circ_0004140 overexpression group was obviously higher than the control group through IHC analysis (Fig. 6C, D). Importantly, the IHC results revealed the CTL exhaustion of circ_0004140 overexpression treatment, which suggested that circ_0004140 may exert the immunosuppressive role through enhancing CCL22 expression (Fig. 6C, E). To further determine the effect of circ_0004140 on immune resistance to anti-PD-1 therapy, we performed the treatment effect of C-021 (CCL22 inhibitor) and anti-PD-1 combination according the dosing regimen (Fig. 6F). The results showed that the combination therapy remarkably suppressed the tumor growth compared with C-021 or anti-PD-1 administration alone (Fig. 6G) and promoted the overall survival rate (Fig. 6H). Taken together, these results revealed that the inhibitor of circ_0004140/CCL22 axis might be a promising potential therapeutic target for anti-PD-1 treatment on LUAD patients.

## Discussion

CircRNAs are pivotal regulators in gene expression on the post-transcriptional level [25]. Increasing evidences reveal that circRNAs are dysregulated in multiple cancers and mediated in the tumorigenesis and metastasis [26, 27]. But the biological function and underlying mechanisms of circRNAs in LUAD still remain elusive. In our research, we firstly identified a novel circRNA (circ_0004140) derived from oncogene YAP1 that plays significant roles in LUAD. Circ_0004140 originate from exon 4-6 of YAP1 and generated a loop structure through joining the 3′ tail and 5′ cap. The stability of circ_0004140 was confirmed by its stable expression under Rnase R digestion. Besides, circ_0004140 was obviously up-regulated in LUAD clinical samples and LUAD cell lines with poor prognosis. Circ_0004140 knockdown suppressed LUAD cell proliferation, migration, and immune resistance in vitro and in vivo.

Accumulating evidence revealed that circRNAs could act functional roles through targeting miRNAs as competing endogenous RNAs (ceRNAs), resulting in modulating miRNA and downstream genes expression [28]. Herein, we demonstrated that circ_0004140 mainly located in cytoplasm and following the RNA pulldown and luciferase reporter assays confirmed the binding interaction between circ_0004140 and miR-1184. Anti-PD-1 therapy is the main predominant immunotherapeutic strategy for LUAD patients [29], while the immune resistance is a key factor leading to poor prognosis [30]. Previous studies indicated that circRNAs are involved in regulating immune resistance in various cancers through different mechanisms including as a sponge of miRNAs. CircMET enhances the EMT process and promotes immunosuppressive microenvironment in HCC. CircMET mediates HCC progression though snail/DPP4/CXCL10 signaling [31]. Ou, et al. determined the HIF-1A-mediated immune invasion from NK cells-mediated killing function in pancreatic carcinoma (PC). Circ_0000977 regulates HIF-1A-mediated immune escape via sponging miR-153/HIF-1A/ADAM10 axis [32]. Herein, we indicated that up-regulation of circ_0004140 is related to cytotoxic T lymphocyte exhaustion and immunotherapy effect in LUAD via circ_0004140/miR-1184/CCL22 axis.

CCL22, also known as macrophage-derived chemokine (MDC), is encoded by the Recombinant Rat MDC/CCL22 gene which located on chromosome 16 [33]. CCL22 is secreted by tumor cells, dendritic cells, and macrophages and regulates the differentiation, proliferation, and localization of lymphocytes and dendritic cells through binding to the CCR4 receptor on the surface of immune cells [34, 35]. Studies have found that CCL22 is dysregulated in various cancers such as gliomas [36], breast [14], gastric cancers [37] and plays an important role in tumor metastasis and immune system function [38]. In our study, we found that circ_0004140/miR-1184/CCL22 axis mediated cell proliferation, migration, and immune resistance in LUAD. Although the up-regulation of CCL22 induced by circ_0004140 regulates LUAD progression, more mechanistic details about CCL22 are still needed and will be our next research stage.

In conclusion, our findings revealed that circ_0004140 was up-regulated in LUAD on vivo and vitro level. Mechanistically, circ_0004140 sponged miR-1184 to regulate proliferation, migration and immune resistance by affecting the expression of target CCL22. Therefore, our study demonstrated that the inhibitor of circ_0004140/CCL22 axis improves anti-PD-1 therapy efficiency and may be a potential novel therapeutic target of LUAD in future.

## Materials and methods

### Specimens and ethics statement

LUAD tissues and normal tissues specimens were obtained from patients treated in Hubei Cancer Hospital (Wuhan, China) (Table 2). Informed consents were obtained from all patients. All experimental procedures were approved by the Ethics Committee of Hubei Cancer Hospital and in conformity with the Declaration of Helsinki of the World Medical Association. All samples were stored at −80 °C before use.

**Table 2:** Clinical characteristics of LUAD patients according to circ_0004140 expression levels.

Feature	circ_0004140 Low	High	*p* value
Age			
<50	38	41	0.639
≥50	42	39	
Gender			
Male	68	70	0.715
Female	12	10	
Tumor size (cm)			
<5	63	15	0.002^**^
≥5	17	65	
Tumor differentiation			
I/II	54	22	0.005^**^
III/IV	26	58	
Lymphatic metastasis			
Yes	21	55	0.011^*^
No	59	25	
Distant metastasis			
Yes	19	57	0.003^**^
No	61	23	

*p < 0.05, **p < 0.01 in statistics.

### Cell culture

Four LUAD cell lines (A549, SPCA-1, NCI-H446, NCI-H292) and BEAS-2B cell line were purchased from TongPai (Shanghai) Biotechnology (Shanghai, China). All cells were maintained in RPMI-1640 medium (Invitrogen, MA, USA) supplemented with 10% FBS (Invitrogen, MA, USA) and 100 U/mL penicillin-streptomycin (Hyclone, MA, USA). The cells incubation was performed at 37 °C in humidified atmosphere containing 5% CO_2_. All cell lines were authenticated using STR profiling and tested negative for mycoplasma contamination.

### Cell transfection

Specific oligonucleotides and plasmids were designed to regulate the expression of circ_0004140, miR-1184, and CCL22. Specific siRNAs targeting circ_0004140 were synthesized from GenePharma (Shanghai, China) and the sequences were listed in Table S1. The full length of circ_0004140, miR-1184, and CCL22 genes were cloned into pLX2 and pVL3 overexpression vectors respectively. The lentiviruses were constructed and packaged by GenePharma (Shanghai, China). MiR-1184 mimics and mimic control were purchased from Sangon Biotech (Shanghai, China) (Table S2). Cell transfection and co-transfection were carried out by Lipofectamine 2000 (Invitrogen, MA, USA) according to the manufacturer’s instructions.

### CCK-8

Cell proliferation was detected via CCK-8 assay (Beyotime, Shanghai, China). 1 × 10^4^ cells were plated on 96-well plates. 10 µL CCK-8 regent was added into each well followed by another 2 h incubation. The absorbance at 450 nm was measured by a microplate reader (Thermo Fisher Scientific, MA, USA). All tests were carried out in triplicate.

### Transwell assay

Transwell chambers coated with matrigel (40 µL of 2 mg/mL) were used to assess the invasion ability of A549 and SPCA-1 cells. After transfection, 1 × 10^5^ of cells suspended in 200 μL RPMI-1640 medium were plated on the upper transwell chamber (Corning, NY, USA). RPMI-1640 medium containing 20% FBS was added into the lower chambers. Incubated 48 h at 37 °C, the cells on the surfaces of bottom chambers were fixed with 20% methanol for 10 min and dyed with 1% crystal violet (Beyotime, Shanghai, China). Finally, photographs were taken using a microscope (Leica, Wetzlar, Germany).

### Wound healing

Cells were cultured in 6-well plates for 24 h with serum-free medium. A wound was created by a sterile pipette tip (200 μL). Cell migration was photographed at 0 and 48 h after scratching under microscope (Leica, Wetzlar, Germany). The total wound healing ratio was analyzed by ImageJ software (Tree Star Inc.).

### Flow cytometric assay of cell apoptosis

Cell apoptosis assay was carried out through an Annexin V-FITC/PI kit (Beyotime, Shanghai, China). After cell transfection, the cells were centrifuged to eliminate the supernatant. Cells were added with 500 μL loading buffer, 5 μL Annexin V-FITC and 10 μL PI solution followed reaction for 20 min according to the manufacturer’s protocol. And the cell apoptosis was obeserved by flow cytometry (BD Biosciences).

### Real-time quantitative PCR

Total RNA was seperated from tissues and cells using TRIzol reagent (Invitrogen, MA, USA) following the manufacturer’s protocol. Then the RNA was reversely transcribed into cDNA using a PrimeScript RT Reagent Kit (Takara, Dalian, China). Real-time qPCR analysis was conducted using a SYBR-Green real-time PCR mixes (Takara, Dalian, China) at an Agilent Mx3005P real-time PCR system (Agilent Technologies). All primers used for qRT-PCR were designed and synthesized by Sangon Biotech (Shanghai, China). GAPDH and U6 were used as internal controls for the mRNA/circRNA and miRNA analysis respectively. The relative expression of genes was calculated by the 2−ΔΔCt method. All reactions were performed in triplicate. The primers used for real-time PCR were as listed in Table. S3.

### Western blot

The total protein was extracted from A549 and SPCA-1 cells by RIPA lysis buffer (Beyotime, Shanghai, China). The total protein concentration was determined using BCA protein assay Kit (Beyotime, Shanghai, China). 20 μg proteins were separated using 10% SDS-PAGE gels, followed by transfer to PVDF membranes (Merk, Darmstadt, Germany). Subsequently, the blots were blocked in 5% skimmed milk and then incubated at 4 °C overnight with primary antibodies including CCL22 (1:1000, Affinity, Shanghai, China) and GAPDH (1:1000, Affinity, Shanghai, China). After incubation with secondary antibody for 4 h at room temperature. The bands were visualized using ECL reagents (Pierce, MA, USA) and imaged by the FluorChem imaging system (BioRad Lab, CA, USA). GAPDH was used as the internal control.

### Fluorescence in situ hybridization (FISH)

FISH assay was carried out to determine the colocalization of circ_0004140 and miR-1184. Briefly, the DNA probe targeting the end-to-head junction of circ_0004140 was labeled with cy3, while the probe of miR-1184 labeled with FITC. Probes were mixed with pre-made hybridization buffer and then samples were incubated in hybridization buffer at 37 °C overnight. Finally, cell nuclei were stained with DAPI and captured using confocal laser-scanning microscopy (Zeiss, Jena, Germany).

### RNA pulldown assay

The biotin-labeled miR-1184 probes and negative control were designed and synthesized by Sango Biotech (Shanghai, China). The miR-1184 probes were incubated with C-1 magnetic beads (Life Technologies, MA, USA) at 25 °C for 2 h to obtain probe-coated beads. A total of 1 × 10^7^ cells were fixed using 1% formaldehyde and then lysed and sonicated. The miR-1184 probes or negative control were incubated with the cell supernatant at 25 °C for 4 h. After washing with wash buffer, the pulldown RNA complex was extracted with a RNeasy Mini Kit (QIAGEN) and subjected to RT-PCR analysis.

### Immunohistochemistry (IHC)

The xenografts tumor tissues were collected, dewaxed in xylene and rehydrated in a series of ethanol. Immunohistochemistry was carried out according to the instructions as described before [39]. Paraffin sections were incubated with primary antibodies against CCL22 (1:500, Affinity, Shanghai, China) or CD8 (1:100, Abcam, Shanghai, China) at 4 °C overnight, followed by incubation with goat anti-mouse horseradish peroxidase (1:2000, Beyotime, Shanghai, China) for 1 h at room temperature. Then the sections were stained with DAB and counterstained with hematoxylin. Finally, photographs were taken using microscope (Leica, Wetzlar, Germany) and the positive staining area was calculated through ImageJ software.

### Dual-luciferase reporter assay

The wild-type (WT)-circ_0004140, Mutant-type (MUT)-circ_0004140, WT-3′UTR-CCL22, and MUT-3′UTR-CCL22 were cloned into pmirGLO reporter vector to obtain luciferase reporter plasmids (Sangon Biotech, Shanghai, China). The plasmids and miR-1184 mimics or negative control were co-transfected into A549 or SPCA-1 cells. After 48 h transfection, cells were harvested and analyzed through a Dual-Luciferase Reporter Assay kit (Promega, CA, USA). The relative luciferase activity was detected through a GloMax fluorescence reader (Promega, CA, USA). Renilla luciferase activity was used to normalize the differences in transfection efficiency. Each assay was carried out in triplicate at least.

### Xenograft tumorigenesis

Five-week-old female BALB/c nude mice were purchased from the Nanjing Biomedical Research Institute of Nanjing University (Nanjing, China). The animals were randomly allocated into groups. A549 cells (4 × 10^5^) stably expressing circ_0004140 or control vector were subcutaneously injected into either side of the back of each mouse. Tumor size was monitored and the mice were sacrificed at the end of treatment (day 40), dissected and weighed. We also performed anti-PD-1 therapy study to evaluate the effect of combination treatment of C-021(CCL22 inhibitor) and anti-PD-1. When the tumor size reached approximately 100 mm^3^, the mice were injected in the tail vein with a PD-1 monoclonal IgG antibody (Hengrui Medicine Company, Lianyungang, China) at 100 μg per dose, or C-021 (Tocris, Bristol, UK) at 5 mg/kg dose [40], or combination injection of anti-PD-1 and C-021. Animal experiments were performed according to the Guide for the Care and Use of Laboratory Animals.

### Statistical analysis

All data analyses were interpreted by GraphPad prism 7.0 version (GraphPad Software, CA, USA). The results are shown as mean ± standard deviation (SD). Statistical analyses were assessed through Student’s *t* test or one-way ANOVA. Pearson analyses were carried out to assess the correlation. Statistical significance was set at *P* < 0.05. There is no estimate of variation in each group of data and the variance is similar between the groups. No statistical method was used to predetermine sample size. The investigators were not blinded to allocation during experiments and outcome assessment. All data were expected to have normal distribution. None of the samples/animals was excluded from the experiment.

## Supplementary information

Supplemental Material

Original, uncropped images of WB

## Data Availability

The datasets used and/or analyzed during the current study are available from the corresponding author on reasonable request.
