# Supplementary material for: circ_0004140 promotes LUAD tumor progression and immune resistance through circ_0004140/miR-1184/CCL22 axis
Source: Cell Death Discov. 2022 Apr 8;8:181. doi: 10.1038/s41420-022-00983-w (PMC8993797; doi:10.1038/s41420-022-00983-w)
Supplement: Supplementary file 1 — Supplemental Material [file 41420_2022_983_MOESM1_ESM.docx]

**Table S1 s**iRNA sequences used in this study.

| **siRNAs** |  |
| --- | --- |
| hsa_circ_0004140-1 | 5′- ATGTCAGGTCCTCTTCCTGAT-3′ |
| hsa_circ_0004140-2 | 5′-AAAATGTCAGGTCCTCTTCCT-3′ |
| hsa_circ_0004140-3 | 5′-AATGTCAGGTCCTCTTCCTGA-3′ |
| si-NC | 5′-CACGATAAGACAATGTATTT-3′ |

**Table S2** miRNA mimics sequences used in this study.

| **mimics** |  |
| --- | --- |
| miR-1184 mimics sense | 5’-CUGCAGCGACUUGAUGGCUUC-3’ |
| miR-1184 mimics antisense | 5’- AAGCCAUCAAGUCGCUGCAGG-3’ |
| mimics control sense | 5’-UUCUCCGAACGUGUCACGUTT-3’ |
| mimics control antisense | 5’-ACGUGACACGUUCGGAGAATT-3’ |

**Table S3** Primers used for real-time PCR in this study.

| **Primer sequence** |  |  |
| --- | --- | --- |
| circ_0004140 | Forward | 5ʹ- CAGCACAGCAAATTCTCCAA -3ʹ |
|  | Reverse | 5ʹ- TTGGGTCTAGCCAAGAGGTG -3ʹ |
| miR-1184 | Forward | 5ʹ- CCTGCAGCGACTTGATGG -3ʹ |
|  | Reverse | 5ʹ- CAGTGCGTGTCGTGGAGT -3ʹ |
| CCL22 | Forward | 5ʹ- CCGTCTGCCCCTGCG -3ʹ |
|  | Reverse | 5ʹ- CACGCCAGGCCTCGG -3ʹ |
| GAPDH | Forward | 5ʹ- CGGTAGAGCGGCCGC-3ʹ |
|  | Reverse | 5ʹ- GGCAGGAGCGCAGGG -3ʹ |
| U6 | Reverse | 5ʹ- AAGATTAGCATGGCCCCTGC-3ʹ |
|  | Forward | 5ʹ- CCATTTCTCGATTTGTGCGTGT-3ʹ |


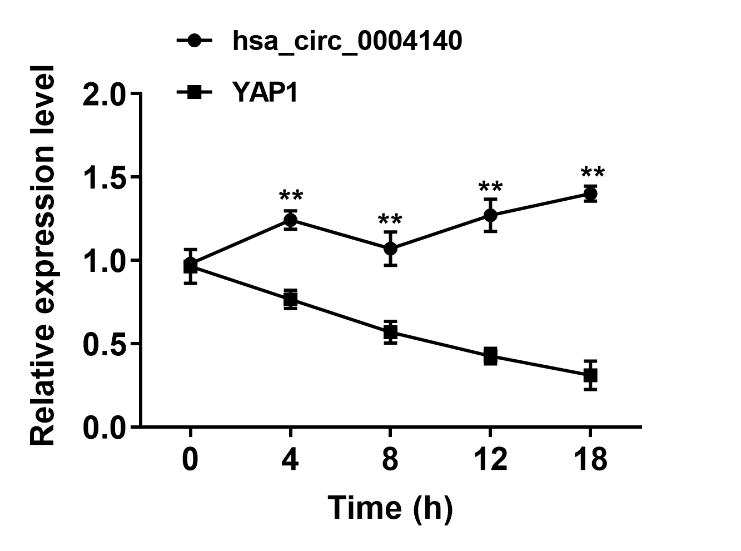


**Fig. S1.** RT-PCR analysis of YAP1 mRNA and circ_0004140 in A549 cells after treatment with Actinomycin D for 0, 4, 8, 12, 18h, respectively (n=3). Data are presented as mean ± SD. **p*<0.05, ***p*<0.01.


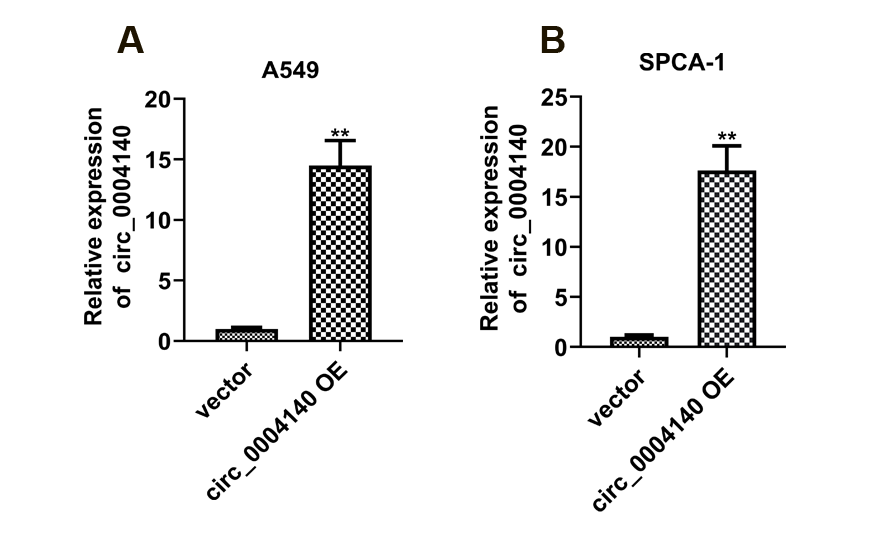


**Fig. S2**. The overexpression efficiency of circ_0004140 overexpression lentiviruses was validated by and RT-PCR in A549 (A) and SPCA-1(B) cells. (n=5). Data are presented as mean ± SD. **p*<0.05, ***p*<0.01.
