# Supplementary material for: circ_0004140 promotes LUAD tumor progression and immune resistance through circ_0004140/miR-1184/CCL22 axis
Source: Cell Death Discov. 2022 Apr 8;8:181. doi: 10.1038/s41420-022-00983-w (PMC8993797; doi:10.1038/s41420-022-00983-w)
Supplement: Supplementary file 2 — Original, uncropped images of WB [file 41420_2022_983_MOESM2_ESM.pdf]

Supplementary Materials:

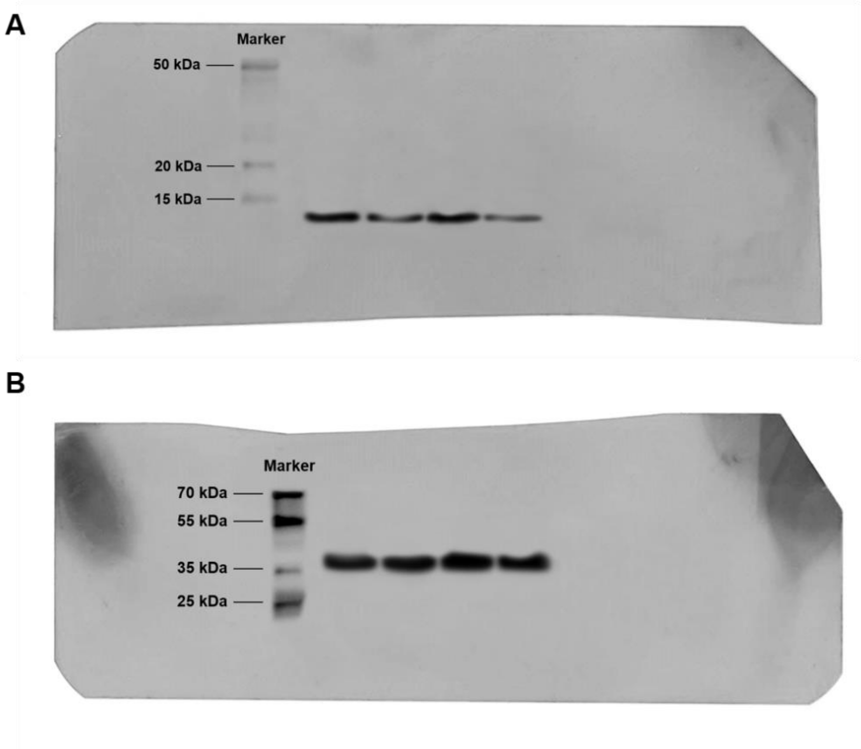

Figure S3. (A-B) Original, uncropped and unadjusted images of Figure 4D. (A) CCL22. (B) GAPDH.

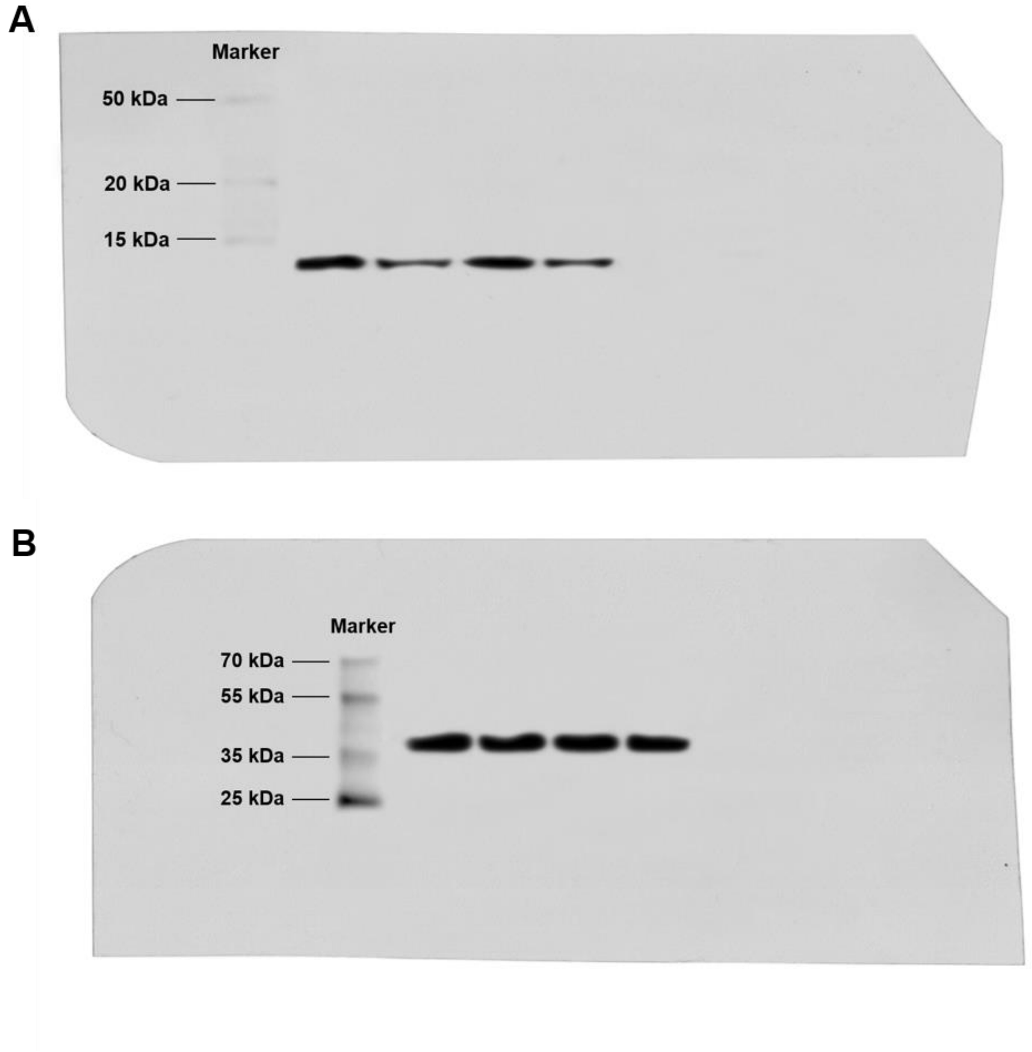

Figure S4. (A-B) Original, uncropped and unadjusted images of Figure 4E. (A) CCL22. (B) GAPDH.
